# Molecular alterations and clinical prognostic factors in resectable non-small cell lung cancer

**DOI:** 10.1186/s12885-024-11934-2

**Published:** 2024-02-13

**Authors:** T. Thamrongjirapat, D. Muntham, P. Incharoen, N. Trachu, P. Sae-Lim, N. Sarachai, K. Khiewngam, N. Monnamo, N. Kantathut, M. Ngodngamthaweesuk, T. Ativitavas, P. Chansriwong, C. Nitiwarangkul, R. Ruangkanchanasetr, A. Kositwattanarerk, E. Sirachainan, T. Dejthevaporn, T. Reungwetwattana

**Affiliations:** 1 Division of Medical Oncology, Department of Medicine, Faculty of Medicine Ramathibodi Hospital, Mahidol University, Bangkok, Thailand; 2 Ramathibodi Lung Cancer Consortium (RLC), Faculty of Medicine Ramathibodi Hospital, Mahidol University, Bangkok, Thailand; 3 Department of Mathematics, Faculty of Science and Technology, Rajamangala University of Technology Suvarnabhumi, Bangkok, Thailand; 4 Department of Pathology, Faculty of Medicine Ramathibodi Hospital, Mahidol University, Bangkok, Thailand; 5 Research Center, Faculty of Medicine Ramathibodi Hospital, Mahidol University, Bangkok, Thailand; 6 Department of Medicine, Faculty of Medicine Ramathibodi Hospital, Mahidol University, Bangkok, Thailand; 7 Division of Thoracic Surgery, Department of Surgery, Faculty of Medicine Ramathibodi Hospital, Mahidol University, Bangkok, Thailand; 8 Division of Diagnostic Radiology, Department of Diagnostic and Therapeutic Radiology, Faculty of Medicine Ramathibodi Hospital, Mahidol University, Bangkok, Thailand; 9 Radiation and Oncology Unit, Department of Diagnostic and Therapeutic Radiology, Faculty of Medicine Ramathibodi Hospital, Mahidol University, Bangkok, Thailand; 10 Division of Nuclear Medicine, Department of Diagnostic and Therapeutic Radiology, Faculty of Medicine Ramathibodi Hospital, Mahidol University, Bangkok, Thailand

**Keywords:** *EGFR* mutation, Resectable NSCLC, Molecular alterations, Prognostic factors

## Abstract

**Background:**

EGFR inhibitor and immunotherapy have been approved for adjuvant treatment in resectable non-small cell lung cancer (NSCLC). Limited reports of molecular and clinical characteristics as prognostic factors in NSCLC have been published.

**Methods:**

Medical records of patients with resectable NSCLC stage I–III diagnosed during 2015–2020 were reviewed. Real time-PCR (RT-PCR) was performed for *EGFR* mutations (*EGFRm*). Immunohistochemistry staining was conducted for *ALK* and PD-L1 expression. Categorical variables were compared using chi-square test and Fisher’s exact test. Survival analysis was done by cox-regression method.

**Results:**

Total 441 patients were included. The prevalence of *EGFRm*, *ALK* fusion, and PD-L1 expression were 57.8%, 1.9%, and 20.5% (SP263), respectively. The most common *EGFRm* were *Del19* (43%) and *L858R* (41%). There was no significant difference of recurrence free survival (RFS) by *EGFRm* status whereas patients with PD-L1 expression (PD-L1 positive patients) had lower RFS compared to without PD-L1 expression (PD-L1 negative patients) (HR = 1.75, *P* = 0.036). Patients with both *EGFRm* and PD-L1 expression had worse RFS compared with *EGFRm* and PD-L1 negative patients (HR = 3.38, *P* = 0.001). Multivariable analysis showed higher CEA at cut-off 3.8 ng/ml, pT4, pN2, pStage II, and margin were significant poor prognostic factors for RFS in the overall population, which was similar to *EGFRm* population (exception of pT and pStage). Only pStage was a significant poor prognostic factor for PD-L1 positive patients. The predictive score for predicting of recurrence were 6 for all population (63% sensitivity and 86% specificity) and 5 for *EGFRm* population (62% sensitivity and 93% specificity).

**Conclusion:**

The prevalence and types of *EGFRm* were similar between early stage and advanced stage NSCLC. While lower prevalence of PD-L1 expression was found in early stage disease. Patients with both *EGFRm* and PD-L1 expression had poorer outcome. Thus PD-L1 expression would be one of the prognostic factor in *EGFRm* patients. Validation of the predictive score should be performed in a larger cohort.

**Supplementary Information:**

The online version contains supplementary material available at 10.1186/s12885-024-11934-2.

## Introduction

Lung cancer has the third highest incidence rate (18.9%) and the highest mortality rate (15.9%) among all cancers in Thailand [1]. Most non-small cell lung cancer (NSCLC) patients (70–80%) present with locally advanced and advanced stage disease; only 20–30% of patients present with early-stage disease [2–4]. Surgery with curative aim is the mainstay treatment for early-stage lung cancer. Despite treatment, the 5-year recurrence rate is still high: 45% for stage IB, 62% for stage II, and 76% for stage III [5]. Adjuvant chemotherapy plays an important role in decreasing the rate of recurrence and death in NSCLC. Previous studies showed an 8%–15% absolute 5-year overall survival (OS) benefit with adjuvant chemotherapy in stage II–IIIA disease or stage I with tumor size ≥ 4 cm (American Joint Committee on Cancer (AJCC) TNM staging system, 6th edition) [6–8]. Furthermore, the Lung Adjuvant Cisplatin Evaluation meta-analysis showed a significant absolute 5-year OS benefit of 5.4% with cisplatin-based chemotherapy, and subgroup analysis demonstrated OS benefit only in stage II–III NSCLC [9].

Epidermal growth factor receptor mutation (*EGFRm*) has been proven as the predictive factor for *EGFR* tyrosine kinase inhibitors (EGFR-TKIs) treatment in advanced NSCLC with *EGFRm.* EGFR-TKIs are an effective treatment and provide a significant increase in survival and better quality of life in *EGFRm *advanced NSCLC patients [10, 11]. The recent “ADAURA” clinical study in early-stage *EGFRm* NSCLC showed a significant increase in disease-free survival (DFS) in patients with stage IB–IIIA who received osimertinib, third generation of EGFR-TKIs, for 3 years as adjuvant treatment after curative resection and/or adjuvant chemotherapy compared with patients who received placebo. DFS was not reached in the osimertinib arm, and DFS was 28.1 months for the placebo arm with a hazard ratio of 0.21 (*P* < 0.001). Thus, osimertinib has become the standard adjuvant treatment for *EGFRm *patients [12]. The prevalence of *EGFRm* in Asian patients with NSCLC advanced disease is approximately 50%–60%; the prevalence of *EGFRm *in early-stage disease is limited [13–17]. Only one Chinese study shown prevalence of 53.6% in early-stage *EGFRm *patients [18]. Clinical studies of adjuvant treatment for other targetable genes are ongoing.

Immunotherapy has become a standard and effective treatment in *EGFR *wild-type NSCLC. Many studies showed significantly better progression-free survival and OS in advanced stage disease treated with immunotherapy compared with doublet platinum-based chemotherapy [19–21]. However, the benefit depends on programmed cell death ligand 1 (PD-L1) expression status; patients with higher level of PD-L1 expression had higher survival benefit. The Impower-010 study recently reported the efficacy of adjuvant atezolizumab after complete resection and complete adjuvant chemotherapy in stage II–IIIA patients with PD-L1 expression. Subgroup analysis also showed better efficacy in patients with high level of PD-L1 expression [22]. Adjuvant atezolizumab has become a new adjuvant treatment option for *EGFR* and *ALK *wild-type patients with early-stage NSCLC. Moreover, other adjuvant immunotherapy study, PEARL/KN091, had also shown benefit in disease free survival for resectable NSCLC stage II-IIIA or IB with tumor size ≥ 4 cm in their reported [23].

These findings highlight the number of currently available adjuvant treatment options for NSCLC patients. However, these treatments are costly for patients. Identifying the patients whom would be benefit from efficient adjuvant treatment is very important, especially for developing countries. In this retrospective study, we explored the prevalence of *EGFRm, ALK* fusion, and PD-L1 expression and clinical characteristics as the prognostic factors in resectable NSCLC.

## Methods

### Patients and study design

**Fig. 1:**
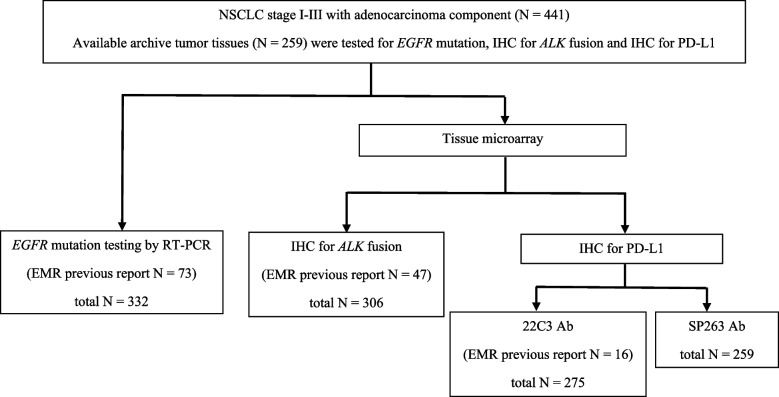
Study flow chart

We retrospectively collected data at Ramathibodi Hospital from electronic medical records of patients with resectable NSCLC stage I–III from January 2015 to December 2020. Patients with age above 18, had adenocarcinoma component in tumor tissue and had curative aim surgery were included, but the patients with pure squamous cell lung cancer and rare types of NSCLC were excluded from this study. Electronic medical record (EMR) for the eligible patients were reviewed for demographic information such as age, sex, performance status, smoking history, clinical and pathological stage by AJCC 8th edition TNM, pathological subtype and adjuvant treatment. We prepared a tumor microarray (TMA) block for available tumor tissue for immunohistochemistry (IHC) staining. In addition, DNA was extracted and examined for 42 *EGFR* mutations. (Fig. 1) This study and all experimental protocols were approved by The Human Research Ethics Committee of Ramathibodi Hospital, Mahidol University, Bangkok, Thailand with the IRB number of COA. MURA2021/682. All methods were carried out in accordance with relevant guidelines and local regulations.

### IHC staining

The Quick-Ray kit (Unitma Co., Ltd. Seoul, Korea) with 2.0 mm in diameter was used to bring out the paraffin-embedded tissues of tumor area. The tissue cores were inserted in a 2 mm recipient block in an array pattern. The block was then embedded to the Plastic Embedding Cassettes. The TMA block was sectioned using a microtome for IHC analysis.

Primary antibodies against *ALK* (D5F3) and PD-L1 (22C3 and SP263 clone) were used for IHC. The concentration and incubation time of samples with antibody was determined following the manufacturer’s recommendations. IHC results were interpreted by a pathologist with IHC expertise. ALK expression is defined as the presence of strong granular cytoplasmic staining in tumor cells (any percentage of positive tumor cells), while PD-L1 expression is defined as the percentage of viable tumor cells showing partial or complete membrane staining at any intensity (Tumor Proportion Score; TPS). A PD-L1 expression level of ≥ 1% is considered as a positive result.

### Amplified Refractory Mutation System (ARMS RT-PCR) assay

Ten formalin-fixed paraffin-embedded (FFPE) sections. (3 μm thickness) were deparaffinized. Genomic DNA was extracted from FFPE tissue using the High Pure FFPET DNA Isolation Kit (Roche Molecular System, Inc.). The DNA sample was tested by the Super-ARMS^Ⓡ^ *EGFR* Mutations Detection Kit (Amoy Diagnostics, Xiamen, China), which has received National Medical Products Administration approval for clinical usage in mainland China. This assay detects 42 *EGFR* mutations in exons 18, 19, 20, and 21. The mutation analysis was performed following the manufacturer’s protocol and using the SLAN-96S real-time PCR system (Shanghai Hongshi Medical Technology Co., Ltd, China). The result was interpreted as positive or negative as defined by the manufacturer’s instructions.

### Statistical analysis

Baseline characteristics, CEA, surgical procedure and tumor characteristics were reported as descriptive variables. Categorical variables were compared using chi-square test and Fisher’s exact test. The prevalence rates of *EGFR* mutation, *ALK* fusion and PD-L1 expression were calculated and summarized as percentages. Recurrence-free survival (RFS) was defined as the time from the date of tissue diagnosis to date of recurrence or death from any causes or to the last follow-up date. OS was defined as the time from date of tissue diagnosis to the date of death from any causes or to the last follow-up date. The status of living or dead was checked with the National Security Death Index of Thailand. RFS and OS were estimated using Kaplan–Meier analysis and compared by stratified log-rank test. The last follow-up date, the living status, and the recurrence status of each patient in electronic medical record was the censor for the survival analysis. The cut-off date for collecting data was on 15 January, 2022. Univariate and multivariate analyses to evaluate prognostic factors for RFS were tested in a Cox-regression model with a level of significance of < 0.05. Predictive score was determined by using hazard ratio from multivariate analysis and receiver operating characteristic curve (ROC curve) analysis to calculate the score. Data were analyzed using Stata version 17.

## Results

### Baseline characteristics

A total of 441 patients were diagnosed with resectable lung adenocarcinoma during 2015–2020 at Ramathibodi Hospital, Thailand. The baseline characteristics are listed in Table 1. There were 332 available tissue samples for *EGFR* mutation testing. For IHC staining of TMA, there were 275, 259, and 306 available tissue samples for analysis of PD-L1 expression (22C3 Ab), PD-L1 expression (SP263 Ab), and *ALK* expression (D5F3 Ab), respectively.

**Table 1:** Baseline clinicopathological characteristics categorized by overall population, *EGFR*, and PD-L1 (SP263) status

Characteristics	Overall population (*n* = 441)	*EGFR* (*n* = 332)		*P* value	PDL1 SP263 (*n* = 259)		*P* value
		Negative (*n* = 140)	Positive (*n* = 192)		Negative (*n* = 206)	≥ 1 (*n* = 53)	
Sex							
Male	160 (36%)	63 (44%)	56 (30%)	0.007	63 (31%)	24 (45%)	0.043
Female	281 (64%)	80 (56%)	133 (70%)		143 (69%)	29 (55%)	
Age, years							
< 65	137 (31%)	41 (29%)	62 (32%)	0.559	61 (30%)	19 (36%)	0.381
≥ 65	304 (69%)	99 (71%)	130 (68%)		145 (70%)	34 (64%)	
COPD	36 (8%)	20 (14%)	8 (4%)	0.001	11 (5%)	8 (15%)	0.033
Smoking							
Never	212 (48%)	56 (40%)	104 (54%)	< 0.001	108 (52%)	23 (43%)	0.085
Former/Current	123 (28%)	54 (39%)	36 (19%)		47 (23%)	20 (38%)	
ECOG							
0–1	339 (77%)	117 (84%)	146 (76%)	0.095	162 (79%)	42 (79%)	0.924
Unknown	102 (23%)	23 (16%)	46 (24%)		44 (21%)	11 (21%)	
CEA							
< 3.8 ng/ml	141 (32%)	47 (34%)	61 (32%)	0.881	71 (16%)	13 (25%)	0.127
≥ 3.8 ng/ml	80 (18%)	24 (17%)	31 (16%)		29 (7%)	13 (25%)	
Surgery procedure							
Lobectomy	377 (85%)	117 (84%)	164 (85%)	0.645	173 (84%)	48 (91%)	0.227
Wedge resection or segmentectomy	64 (15%)	23 (16%)	28 (15%)		33 (16%)	5 (9%)	
Pathology							
Adenocarcinoma	415 (94%)	128 (92%)	187 (97%)	0.017	196 (95%)	51 (96%)	1
Adenosquamous	23 (5%)	9 (6%)	5 (3%)		9 (4%)	2 (4%)	
NSCLC, NOS	3 (1%)	3 (2%)	0		1 (1%)	0	
HIstology subtype							
MIA	13 (3%)	3 (2%)	2 (1%)	< 0.001	3 (1%)	1 (2%)	< 0.001
Lepidic	106 (24%)	26 (19%)	47 (24%)		56 (27%)	5 (9%)	
Acinar	192 (44%)	42 (30%)	104 (54%)		92 (45%)	21 (40%)	
Papillary	38 (9%)	10 (7%)	21 (11%)		20 (10%)	7 (13%)	
Micropapillary	15 (3%)	9 (6%)	5 (3%)		5 (2%)	2 (4%)	
Solid	26 (6%)	15 (11%)	6 (3%)		4 (2%)	9 (17%)	
Mucinous	33 (7%)	24 (17%)	4 (2%)		20 (10%)	3 (6%)	
pT							
T1	246 (56%)	71 (51%)	106 (55%)	0.012	117 (57%)	27 (51%)	0.861
T2a	99 (22%)	24 (17%)	52 (27%)		45 (22%)	14 (26%)	
T2b	31 (7%)	12 (8%)	14 (7%)		14 (7%)	4 (8%)	
T3	52 (12%)	25 (18%)	16 (24%)		25 (12%)	6 (11%)	
T4	13 (3%)	8 (6%)	4 (2%)		5 (2%)	2 (4%)	
pN							
N0	365 (82%)	115 (82%)	150 (78%)	0.756	175 (85%)	34 (64%)	0.003
N1	38 (9%)	11 (8%)	20 (10%)		13 (6%)	6 (11%)	
N2	38 (9%)	14 (10%)	22 (11%)		18 (9%)	13 (25%)	
Margin							
Negative	429 (97%)	135 (96%)	185 (96%)	0.971	199 (97%)	51 (96%)	1
Positive	12 (3%)	5 (4%)	7 (4%)		7 (3%)	2 (4%)	
LVI							
Negative	284 (64%)	84 (60%)	120 (63%)	0.871	141 (68%)	25 (47%)	0.006
Positive	142 (32%)	52 (37%)	66 (34%)		58 (23%)	26 (49%)	
Pleural invasion							
Negative	292 (66%)	88 (63%)	125 (65%)	0.335	132 (64%)	32 (60%)	0.031
Positive	113 (26%)	41 (29%)	45 (23%)		47 (23%)	16 (30%)	
Pathological staging							
I	301 (68%)	80 (57%)	132 (69%)	0.061	145 (70%)	26 (49%)	0.003
II	82 (19%)	37 (27%)	32 (17%)		36 (18%)	11 (21%)	
III	58 (13%)	23 (16%)	28 (14%)		25 (12%)	16 (30%)	
Adjuvant systemic treatment							
Cisplatin-based	45 (10%)	16 (11%)	26 (14%)	0.194	19 (9%)	11 (21%)	0.122
Carboplatin-based	44 (10%)	17 (12%)	15 (8%)		17 (8%)	6 (11%)	
Others	5 (1%)	2 (1%)	3 (2%)		3 (1%)	1 (2%)	
Unknown	3 (1%)	3 (2%)	0		2 (1%)	0	
Adjuvant radiation							
RT alone	2 (0.5%)	2 (1%)	0	0.292	0	2 (4%)	0.005
CCRT	6 (1%)	1 (0.7%)	4 (2%)		4 (2%)	1 (2%)	
Sequential	17 (14%)	8 (6%)	8 (4%)		7 (3%)	6 (11%)	
Recurrent							
Locoregional	20 (5%)	10 (7%)	9 (5%)	0.585	41 (20%)	16 (30%)	0.229
Distant metastasis	99 (22%)	39 (28%)	51 (27%)		9 (4%)	3 (6%)	
CNS metastasis	19 (4%)	6 (4%)	11 (6%)	0.556	5 (2%)	4 (8%)	0.088

*pT* pathological tumor stage, *pN* pathological nodal stage, *LVI* Lymphovascular invasion, *RT* Radiation, *CCRT* Concurrent radiation, *NA* not available

Most patients were female (64%), older than 65 years (69%), and non-smokers (48%); most had good performance status (ECOG 0–1) (77%) and were treated by surgery with lobectomy procedure (85%). Most tumors were pathological stage I (68%), had negative margin (97%), and showed no pleural invasion (66%). Nodal status of N1 and N2 was equally found (approximately 9% for each). Adenocarcinoma was the most frequently detected cell type (94%), whereas the mixed adenosquamous cell was found in only 5%. Acinar subtype was predominant (44%), followed by the lepidic subtype (24%), papillary subtype (9%), mucinous subtype (7%), solid subtype (6%), micropapillary (3%), and minimally invasive adenocarcinoma (3%). Out of 441 patients, 89 (20%) patients received adjuvant doublet platinum-based chemotherapy. The percentages of patients treated with cisplatin-based chemotherapy and carboplatin-based chemotherapy regimens were similar (10%). Of 97 patients who received other adjuvant regimens, 5 patients received gemcitabine alone (1 patient), gefitinib (3 patients) or alectinib (1 patient). Twenty-five patients in the overall group (5.5%) had adjuvant radiation after surgery; these patients received sequential chemotherapy-radiation (4%), concurrent chemoradiation (CCRT) (1%), and radiation (RT) alone (0.5%). In the overall group, 121 patients (27.4%) developed recurrent disease; 99 of the 121 (82%) had distant metastasis disease and 19 patients (15.7%) had central nervous system (CNS) metastasis. The mean time from diagnosis to surgery was 35 days and the mean time from surgery to adjuvant treatment was 45 days.

In the subgroup with tissues available for *EGFR* testing (*N* = 332), gender, chronic obstructive pulmonary disease (COPD) status, smoking status, pathological subtype, and pathological T stage (pT) were significantly different between *EGFRm* patients and *EGFR* wild-type patients (Table 1). Female, never-smoker, acinar/lepidic/papillary subtype, and pT2/pT3 patients were markedly more prevalent in the *EGFRm* group compared with the *EGFR* wild-type group. The *EGFR* wild-type group had significantly more COPD patients. The clinical characteristics of *EGFRm* patients were similar to those of the overall population.

The PD-L1 testing population (*N* = 259; SP263 Ab) had significant differences in gender, COPD status, pathological subtype, pathological lymph node stage (pN stage), lymphovascular invasion (LVI) status, pathological stage, and number of organs involved compared with the overall patient group (Table 1). The prevalence rates of males, COPD patients, former/current smokers, solid/papillary/micropapillary subtypes, pN1/pN2, positive LVI, stage II/III, and ≥ two organ metastases were significantly higher in PD-L1-positive patients compared with PD-L1-negative patients.

Only 6 out of 306 patients (2%) were *ALK* fusion positive, and 5 of the 6 (83%) were younger than 65 years old. The median age of *ALK* fusion positive patients was 55 years old. The disease characteristics of *ALK* fusion positive patients were aggressive; 66% showed N2 disease, 83% had positive LVI, and 66% had pathological stage III disease. All *ALK* fusion positive patients developed recurrent disease and all had distant metastases.

### Prevalence of molecular alterations

The prevalence of *EGFRm* was 57.8%. The prevalence rates of *EGFRm* in each stage were 59.6%, 69.9%, 40%, 49%, 57.5% and 45.5% in stage IA, IB, IIA, IIB, IIIA and IIIB, respectively. The most common *EGFR* mutations were exon 19 deletion *(Del19)* (43%) and *L858R* (41%). Combined mutations patients were 10%. Of the 192 patients, 11 (6%) had mutations between *Del19/T790M and L858R/T790M* and 8 (4%) had combined mutations between *Del19/L858R and G719X/L861Q/S768I* or between *G719X* and *S768I*. Approximately 6% of *EGFRm* patients had uncommon mutations such as *S768I* (1 patient, 0.05%), *G719X* (4 patients, 0.2%), *T790M* (3 patients, 0.15%), and exon 20 insertion (3 patients, 0.15%).

The prevalence of PD-L1 expression with tumor proportion score (TPS) ≥ 1 was 20.5% when evaluated by SP263 Ab and 17.5% when evaluated by 22C3 Ab. The correlation between these two antibodies was tested and showed agreement of 93.4% (kappa = 0.78) which suggested of high-rate agreement [24]. The prevalence of PD-L1 expression (SP263 Ab) and TPS ≥ 1 by stage were 16%, 14%, 14%, 27%, 47%, and 1% in stage IA, IB, IIA, IIB, IIIA, and IIIB, respectively. The prevalence of *ALK* fusion was 2% in the overall population.

### Recurrence-free survival

**Fig. 2:**
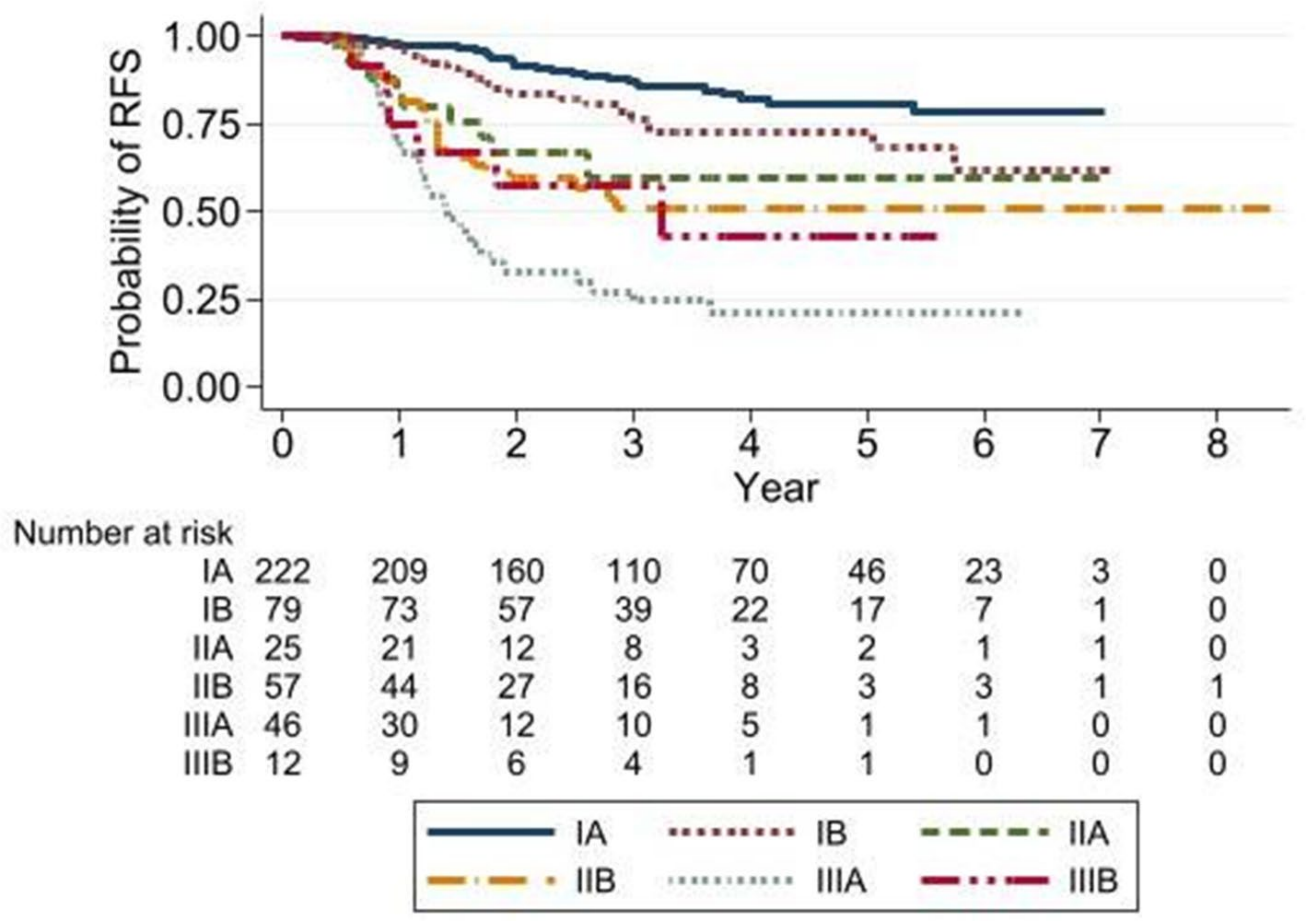
Recurrence-free survival curve in the overall population by stage

In the overall population cohort, 121 (27.4%) had recurrent disease. The 5-year RFS rate was 66% for the entire cohort, and the 5-year RFS rates stratified by stages were 80%, 72%, 59%, 50%, 21%, and 42% for stage IA, IB, IIA, IIB, IIIA, and IIIB, respectively (Table 2, Fig. 2). The 5-year RFS in the *EGFRm* cohort as 66% in stage I patients, which was lower than the rate in the overall population cohort. There was no available data for stage II and III due to none of recurrence and death (events) occurred. The RFS of 1-year to 5-year for each stage were not significantly different between *EGFRm* and *EGFR* wild-type patients. The 5-year RFS in the PD-L1 positive cohort was 82% for stage I, and 20% for stage III, which was similar to the rates in the overall population cohort and not statistically different to PD-L1 negative patients (Table 2). There was no available data in stage II due to no events occurred in this group of patients. Distant metastasis was higher than locoregional recurrence in the overall population cohort (22% vs 5%) and *EGFRm* cohort (27% vs 5%) while the opposite was observed in the PD-L1-positive cohort (6% vs 30%). CNS metastasis was found in 4%, 6%, and 8% in the overall population cohort, *EGFRm* cohort, and PD-L1 positive cohort, respectively. Regarding adjuvant treatment, RFS was not different between patients who received adjuvant treatment and patients who did not receive treatment. There was also no statistical difference in RFS between cisplatin- and carboplatin-based regimens in the overall population cohort (*P* = 0.085).

**Table 2:** Recurrence-free survival rate of the overall population and *EGFRm* and PD-L1 expression cohorts according to stage

Stage	1 year (%)	2 years (%)	3 years (%)	4 years (%)	5 years (%)
Overall population	91	77	71	67	66
IA	97	91	87	82	80
IB	96	83	76	72	72
IIA	84	66	59	59	59
IIB	83	59	50	50	50
IIIA	71	32	27	21	21
IIIB	75	57	57	42	42
*EGFR cohort*	92	71	65	60	58
I/*EGFR*-	94	89	78	73	62
I/*EGFR* +	97	83	77	70	66
II/*EGFR*-	74	52	35	35	35
II/*EGFR* +	90	61	56	56	NA
III/*EGFR*-	73	49	43	26	26
III/*EGFR* +	70	29	23	23	NA
PD-L1 cohort	86	66	62	51	51
I/PDL1-	95	87	82	75	70
I/PDL1 +	100	94	94	82	82
II/PDL1-	88	72	61	61	61
II/PDL1 +	90	58	29	NA	NA
III/PDL1-	78	41	27	20	20
III/PDL1 +	62	31	31	20	20

*NA* not available

**Fig. 3:**
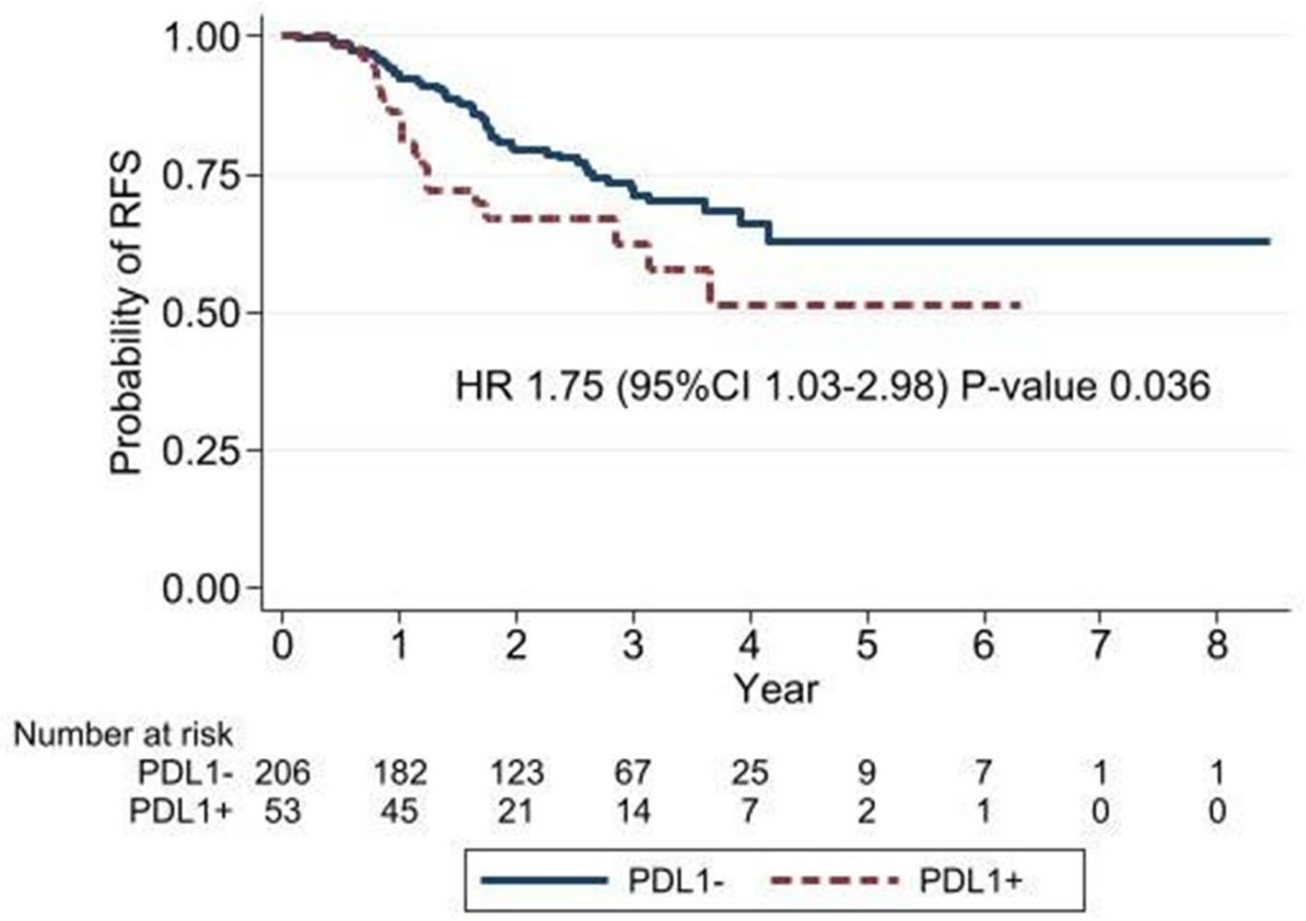
Comparison of recurrence-free survival between PD-L1-positive and -negative patients

**Fig. 4:**
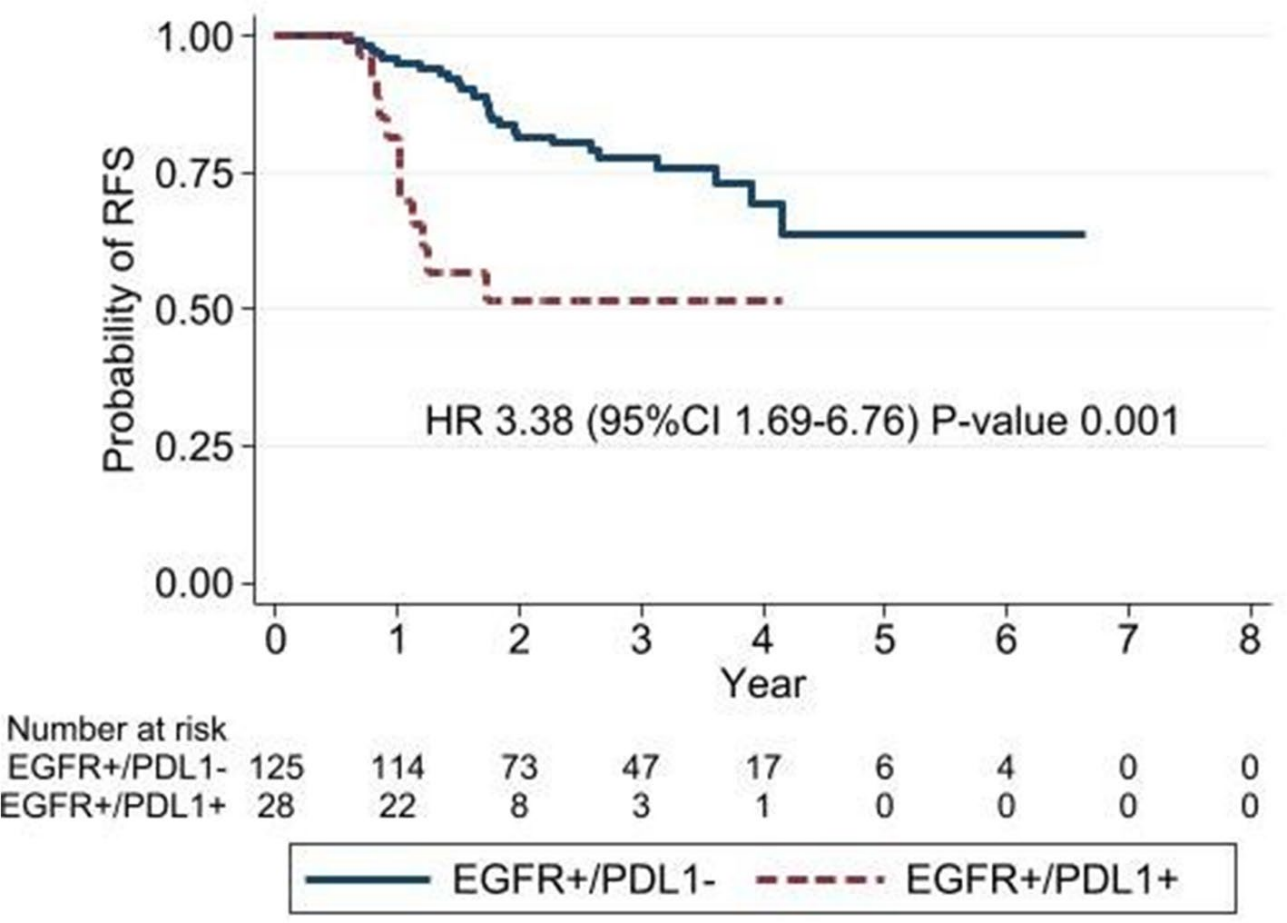
Recurrence-free survival according to PD-L1 expression status in *EGFRm* patients

Interestingly, we found a significantly worse 5-year RFS in PD-L1-positive patients (51%) compared with PD-L1 negative patients (62%) (HR = 1.75, 95%CI 1.03–2.98, *P* = 0.036) (Fig. 3). Patients with both *EGFRm* and PD-L1 expression had significantly worse RFS compared with double-negative patients (HR = 2.21, 95%CI 1.09–4.48, *P* = 0.027). *EGFRm* patients with negative PD-L1 expression also had significantly better RFS compared with *EGFRm* positive and PD-L1 positive patients (HR = 3.38, 95%CI 1.69–6.76, *P* = 0.001) (Fig. 4).

### Overall survival

At the cut-off date (Jan 15th, 2022), 87% of all patients were still alive. The 5-year OS rate was 82%, and the 5-year OS rates were 91%, 89%, 63%, 63%, 58% and 58% in stage IA, IB, IIA, IIB, IIIA, and IIIB, respectively. There was no significant difference in OS by adjuvant systemic chemotherapy in stage II–III (HR = 0.77, 95%CI 0.39–1.50, *P* = 0.453), adjuvant chemotherapy regimen (HR = 0.66, 95%CI 0.26–1.65, *P* = 0.381), and PD-L1 expression status (HR = 1.04, 95%CI 0.339–2.76, *P* = 0.934). *EGFRm* patients had a significantly longer OS compared with *EGFR* wild-type patients (HR = 0.31, 95%CI 0.16–0.57, *P* < 0.001). However, 60/192 (32%) of *EGFRm* patients had recurrent disease and 46 received EGFR-TKIs. Furthermore, there was no statistical difference in OS in PD-L1 status.

### Clinical correlation and prognostic factors

Univariate analysis in the overall population showed that smoking, high CEA (cut-off 3.8 ng/ml), pathological stage II–III, larger tumor size, pN1/pN2, positive margin, positive LVI, positive pleural invasion, non-lepidic pathological subtype and positive PD-L1 by SP263 had a significant poorer outcome for RFS. However, multivariate analysis showed that only higher CEA, pT4, pN2, pathological stage II and margin were significant prognostic factors for RFS (Table 3).

**Table 3:** Univariate analysis by prognostic factors for recurrence-free survival

Prognostic factors	Overall Cohort		*EGFRm* Cohort		PD-L1 positive Cohort	
	**HR (95%CI)**	***P* value**	**HR (95%CI)**	***P* value**	**HR (95%CI)**	***P* value**
**Smoking**						
Never	Ref		Ref		Ref	
Current or former	1.55 (1.03–2.34)	0.033	2.12 (1.15–3.89)	0.015	0.44 (0.15–1.22)	0.117
**COPD**						
No	Ref		Ref		Ref	
Yes	1.72 (0.98–3.01)	0.054	1.39 (0.43–0.46)	0.572	0.51 (0.11–2.23)	0.375
**CEA**						
< 3.8 ng/ml	Ref		Ref		Ref	
≥ 3.8 ng/ml	2.57 (1.57–4.19)	< 0.001	4.84 (2.23–10.46)	< 0.001	1.75 (0.55–5.56)	0.339
**pT**						
pT1	Ref		Ref		Ref	
pT2a	2.02 (1.27–3.20)	0.003	1.91 (1.05–3.46)	0.031	2.44 (0.82–7.29)	0.108
pT2b	3.73 (2.03–6.85)	< 0.001	3.80 (1.62–8.97)	0.002	0.94 (0.11–7.92)	0.961
pT3	3.81 (2.30–6.30)	< 0.001	2.22 (0.95–5.19)	0.064	3.90 (1.09–13.94)	0.036
pT4	4.98 (2.42–10.24)	< 0.001	4.73 (1.41–15.83)	0.012	2.16 (0.25–18.29)	0.477
**pN**						
pN0	Ref		Ref		Ref	
pN1	3.88 (2.33–6.44)	< 0.001	2.93 (1.48–5.78)	0.002	2.01 (0.40–10.07)	0.395
pN2	4.31 (2.18–8.53)	< 0.001	5.69 (3.08–10.51)	< 0.001	7.43 (2.72–20.31)	< 0.001
**pStage**						
I	Ref		Ref		Ref	
II	3.30 (2.12–5.13)	< 0.001	2.15 (1.11–4.17)	0.022	7.16 (1.37–37.24)	0.019
III	6.74 (4.40–10.33)	< 0.001	6.53 (3.65–11.69)	< 0.001	14.27 (3.18–64.02)	0.001
**Margin**						
Negative	Ref		Ref		Ref	
Positive	4.31 (2.18–8.53)	< 0.001	3.81 (1.52–9.59)	0.004	1.44 (0.18–11.02)	0.724
**LVI**						
Negative	Ref		Ref		Ref	
Positive	3.28 (2.27–4.74)	< 0.001	3.22 (1.90–5.43)	< 0.001	5.96 (1.73–20.51)	0.005
**Pleural invasion**						
Negative	Ref		Ref		Ref	
Positive	2.35 (1.61–3.42)	< 0.001	2.18 (1.28–3.71)	0.004	1.58 (0.59–4.18)	0.354
**Histology**						
Lepidic	Ref		Ref		Ref	
Acinar	2.59 (1.46–4.60)	0.001	3.54 (1.49–8.42)	0.004	0.91 (0.19–4.32)	0.908
Micropapillary	5.41 (2.29–12.78)	< 0.001	4.33 (0.87–21.54)	0.073	1.25 (0.23–6.72)	0.789
Papillary	4.15 (2.02–8.50)	< 0.001	4.58 (1.62–12.94)	0.004		1
Solid	4.86 (2.27–10.40)	< 0.001	10.66 (2.97–38.22)	< 0.001	0.45 (0.072–2.85)	0.401
Mucinous	2.83 (1.27–6.30)	< 0.001	6.69 (1.33–33.49)	0.021	0.73 (0.06–8.10)	0.801
***EGFR* status**						
Wild-type	Ref		-		-	
Mutated	0.88 (0.60–1.28)	0.518	-		-	
**PDL1 SP263**						
Negative	Ref		-		-	
≥ 1	1.75 (1.03–2.98)	0.036	-		-	

*HR* Hazard ratio, *Ref* Reference

In the *EGFRm* population, high CEA (cut-off 3.8 ng/ml), pathological stage II–III, larger tumor size, pN1/pN2, positive margin, positive LVI, positive pleural invasion, non-lepidic and non-micropapillary subtype were significantly worse clinical factors for RFS in univariate analysis. Nevertheless, only high CEA, pN1 and positive margin were significant factors in multivariate analysis in this population. Only pathological stage was a poor prognostic factor for PD-L1-positive patients in multivariate analysis (Table 3).

### Predictive score

We proposed a predictive score (Table 4) for predicting recurrent disease after curative surgery using significant clinical factors in univariate and multivariate analyses. We also added some significant clinical factors in our score because they were considered as strong clinical factors in previous studies [25]. We proposed score in 2 populations which were all population and *EGFRm* population. Because of the low number of PD-L1-positive patients, we could not analyze the predictive score in this cohort.

**Table 4:** Predictive score for predicting recurrent disease

Factors	Score	
	**Overall Population**^a^	***EGFR***^b^
**CEA**		
< 3.8 ng/ml	0	0
≥ 3.8 ng/ml	2	3
**pT**		
pT1/2a	0	0
pT2b	1	1
pT3	2	1
pT4	18	34
**pN**		
pN0	0	0
pN1	4	4
pN2	6	6
**Margin**		
Negative	0	0
Positive	2	39
**LVI**		
Negative	0	
Positive	1	
**Pleural invasion**		
Negative	0	0
Positive	1	2
**Histology subtype**		
Lepidic	0	
Acinar	1	
Micropapillary	1	
Papillary	4	
Solid	2	
Mucinous	3	

*pT* pathological tumor stage, *pN* pathological nodal stage, *LVI* Lymphovascular invasion

^a^Cut-off ≥ 6 with sensitivity of 63% and specificity 86% ROC

^b^Cut-off ≥ 5 with sensitivity of 62% and specificity 93% ROC

#### Overall population cohort

The predictive factors were CEA cut-off 3.8 ng/ml, pT, pN, margin, LVI, pleural invasion, and histology subtype. The score for each factor is shown in Table 5. We selected a cut-off point ≥ 6 for determining the risk of recurrent disease with 63% sensitivity and 86% of specificity (ROC = 0.75, Supplement Fig. 1).

**Table 5:** Multivariate analysis by prognostic factors for recurrence-free survival

Prognostic factors	Overall Cohort		*EGFRm* Cohort		PD-L1 Positive Cohort	
	**HR (95%CI)**	***P* value**	**HR (95%CI)**	***P* value**	**HR (95%CI)**	***P* value**
**CEA**						
< 3.8 ng/ml	Ref		Ref		-	-
≥ 3.8 ng/ml	6.31 (1.73–22.9)	0.005	9.62 (2.80–33.02)	< 0.001	-	-
**pT**						
pT1	Ref		-	-	-	-
pT2a	-	-	-	-	-	-
pT2b	0.26 (0.02–3.14)	0.294	-	-	-	-
pT3	0.47 (0.07–3.05)	0.437	-	-	-	-
pT4	253.87 (2.59–24867.46)	0.018	-	-	-	-
**pN**						
pN0	Ref	-	Ref	-	-	-
pN1	0.78 (0.06–8.97)	0.848	5.36 (1.54–18.69)	0.008	-	-
pN2	49.5 (1.02–2393.91)	0.049	2.77 (0.72–10.58)	0.135	-	-
**pStage**						
I	Ref		-	-	Ref	
II	44.01 (2.46–786.99)	0.01	-	-	7.06 (1.35–36.87)	0.02
III	0.710 (0.01–46.55)	0.875	-	-	16.23 (3.61–72.87)	< 0.001
**Margin**						
Negative	Ref		Ref		-	-
Positive	65.05 (4.06–1039.87)	0.003	11.74 (1.99–69.18)	0.006	-	-

*HR* Hazard ratio, *Ref* Reference

#### *EGFRm* population cohort

Higher CEA, pT, pN, margin, and pleural invasion were significant predictive factors for the *EGFRm* population. The scores are shown in Table 5. We selected a cut-off point ≥ 5 for determining risk of recurrent disease with 62% sensitivity and 93% of specificity (ROC = 0.77, Supplement Fig. 2).

## Discussion

This retrospective study in early-stage NSCLC in a Thai population showed a 5-year RFS rate of 66% and 5-year OS rate of 82%, which were better than previously published rates [26]. The 5-year RFS rates were 58% and 51% and 5-year OS rates were 87% and 89% in the *EGFRm* and positive PD-L1 groups, respectively.

Previous studies showed a significantly improved 5-year absolute survival benefit rate (8%–15%) of adjuvant chemotherapy compared with placebo in patients with stage II–III disease [9, 27, 28]. Our data did not show significant improvement of RFS or OS with adjuvant systemic treatment after surgery for all stages of disease because the majority of our patients had stage I disease (68%) and only 22% of all patients received adjuvant chemotherapy. There was no benefit of adjuvant chemotherapy in stage I disease patients in our population, which was similar to results in the CALGB69633 study [29]. Moreover, the cisplatin-based regimen did not show a difference in RFS or OS benefit compared with a carboplatin-based regimen; these results were comparable with previous retrospective data in Canada [30]. Their study used platinum combination with vinorelbine only but in our retrospective study we used various chemotherapy in combination with cisplatin or carboplatin such as etoposide, pemetrexed or paclitaxel. However, it might not be concluded as equal efficacy of cisplatin or carboplatin based chemotherapy due to only single center report. The recurrence rate after complete resection in our study was 27.4%, which was similar to previous reports (20%–40%) [5, 25, 31]. We also found high recurrence in *EGFR* wild-type patients (49/140; 35%), *EGFRm *patients (60/192; 31.3%), PD-L1 positive patients (19/53; 35.8%), and PD-L1 negative patients (50/206; 24.3%). In the PD-L1 positive patients, we found a higher rate of N2 disease, LVI positivity, and pleural invasion, which were our potential worse prognosis factors. This might explain the higher rate of recurrence in the PD-L1 positive population compared to the PD-L1 negative population. Regarding the pattern of recurrence, 82% of recurrent patients developed distant metastasis and 15.7% of recurrent patients had CNS metastasis. A previous report in Poland showed a similar rate of distant metastasis (79.5%) but a higher rate of CNS metastasis (22.9%) [31–33]. Interestingly, we also found a high CNS metastasis rate in *EGFRm* patients who had recurrent disease (11/60; 18.3%) and in PD-L1 positive patients (4/19; 21%). The prevalence of *EGFRm* in early-stage NSCLC in this study was 57.8% and comparable with the *EGFRm *rate in our previous report (60%) and other reports (49%–68%) in metastatic NSCLC in a Thai population [15–17, 34]. In addition, a report from China showed an *EGFRm *rate in early-stage NSCLC of 53.6% which also similar to our study [18]. In this study, the most common *EGFRm* was *Del19* (43%), followed by *L858R* (41%), which was comparable with the previous studies. The prevalence rate of combined mutation (10%) and uncommon mutation (6%) were slightly higher than other studies [35], and we also found 3% of patients with de novo resistance mutation (*T790M *and exon 20 insertion), which was consistent with previous reports in both early and advanced disease [14, 18]. RFS outcome was not affected by *EGFR* status, but there was significantly better OS in *EGFRm* patients (HR 0.31, 95%CI 0.16–0.57, *P* < 0.001) because of EGFR*-*TKI treatment in recurrent patients. Prevalence of positive PD-L1 expression (≥ 1%) in early-stage NSCLC patients in our study was 20.5% (SP263 Ab) and 17.5% (22C3 Ab) which was lower prevalence compared to metastatic NSCLC patients in real world data from multicenter in USA, Canada, Spain, Russia, Denmark, Argentina, Columbia, Japan, and Hong Kong (52%) [35]. Both PD-L1 assays (SP263 and 22C3) had a proven 93.4% correlation of results with kappa 0.78.

Recently data shown that *EGFRm *tumor was probably induced PD-L1 expression on tumor cell which activated immune escape mechanism [36, 37]. Therefore tumors with combined positive of *EGFRm* and PD-L1 had worst survival outcome than *EGFRm* and PD-L1 negative tumor. Furthermore, similar to the previous studies [38–41] which had reported of poorer RFS and OS in advanced stage *EGFRm* NSCLC with positive PD-L1 compared with PD-L1 negative patients. Our result also showed that *EGFRm* and *PD-L1*-positive patients (28/192, 14.6%) showed significantly poorer RFS compared with *EGFRm* and PD-L1-negative patients (125/192, 65%). Thus PD-L1 expression is possibly one of the prognostic factor in *EGFRm* patients and it might be one of the biomarker for selecting the patients whom might have the most benefit from adjuvant EGFR-TKI treatment in the future. Even though we need more data to confirm this hypothesis.

Our results showed that former or current smoker, CEA ≥ 3.8 mg/ml, large tumor size, positive lymph nodes, pathological stage II–III, positive margin or LVI, invasion of pleura, non-lepidic subtype, and positive PD-L1 expression were significantly associated with poorer outcomes in univariate analysis. Only higher CEA, pT4, pN2, positive margin and pathological stage II were significant in multivariate analysis. These outcomes were also consistent with previous retrospective studies [25, 33]. Furthermore, we also found similar poor prognostic factors in the *EGFRm* population in univariate analysis, and only higher CEA, pN1/pN2, and positive margin correlated with significantly worse survival outcome.

We also proposed a predictive score for predicting risk of recurrence in the overall population and *EGFRm* cohort. The sample size was too small to generate a predictive score in the PD-L1-positive cohort. A score ≥ 6 in the overall population cohort showed a greater risk of recurrence of disease with 63% sensitivity and 86% specificity; a score ≥ 5 in the *EGFRm* cohort showed greater risk of recurrence of disease with 62% sensitivity and 93% specificity. This score may help in decision making for selecting the proper adjuvant systemic treatment in high-risk resectable NSCLC. A previous study [42] had proposed a 5-year DFS predictive score in stage I NSCLC. The intermediate to high-risk patients had significantly decreased in 5-year DFS and a higher distant relapse rate compared with low-risk patients. Their prognostic factors including smoking status, malignancy history, resection method, and histology type (adenocarcinoma vs non-adenocarcinoma), which were not significant factors in our study. Another study also proposed a risk score to predict OS in patients of all stages of NSCLC. The predictive score was calculated from stage, NSCLC NOS subtype, no proven actionable mutation, poor ECOG, ever smoker, respiratory comorbidity, weight loss, male and older age, which were different from our study. However, the higher score was also associated with poorer survival outcomes [43].

Regarding the current situation for adjuvant treatment after curative surgery in Thailand, most of the patient could not access to EGFR-TKI and immunotherapy, thus the backbone adjuvant treatment is doublet platinum-based chemotherapy for high-risk patients. Clinicopathological factors as found in our result (CEA level, tumor size, lymph node status, margin) were still important prognostic factors which determine clinical decision for adjuvant treatment in Thailand. However, in era of personalized treatment, adjuvant EGFR-TKI and adjuvant immunotherapy are challenging in management in early stage NSCLC. To gain the knowledge and explore for the new biomarker are important for selecting the patients whom will receive the most benefit from adjuvant EGFR-TKI treatment and adjuvant immunotherapy.

This study has several limitations. Firstly, the data were retrospective and we could not control affecting factors. Secondly, there was some of the missing data. Thirdly, there was a small number of stage II–III patients, who were the majority of patients who received adjuvant treatment. We also had a short follow-up period (mean follow-up time of 44 months), and thus a longer follow-up will be needed in further analysis.

## Conclusion

The prevalence of *EGFR* mutation in the early-stage NSCLC in Thai patients similar to rates reported in advanced stage disease whereas the prevalence of PD-L1 expression was lower than advanced stage disease. The prevalence of *ALK* fusion gene in early-stage NSCLC was quite low in our population. There was no difference in *EGFRm* and PD-L1 prevalence in each stage of disease (I–III). We confirmed a good correlation between 22C3 Ab and SP263 Ab for PD-L1 expression testing. The OS of *EGFRm* patients was significantly longer than the OS of *EGFR* wild-type patients, which might be the effect of EGFR-TKI treatment in recurrent disease. PD-L1 expression might be the crucial prognostic factors for *EGFRm* resectable NSCLC which probably help the clinicians to select the most benefit patients for adjuvant EGFR-TKI treatment in developing countries. Novel biomarkers are important for helping patient selection to receive the most benefit from adjuvant EGFR-TKI treatment and adjuvant immunotherapy. The predictive scores for resectable lung cancer should be explored and validated in a larger cohort.

### Supplementary Information

**Additional file 1: Supplement figure 1**. ROC curve of predictive score of overall population.** Supplement figure 2**. ROC curve of predictive score of *EGFRm* cohort.

## Data Availability

The datasets generated during and/or analyzed during the current study are available from the corresponding author on reasonable request.
