# Supplementary material for: Molecular alterations and clinical prognostic factors in resectable non-small cell lung cancer
Source: BMC Cancer. 2024 Feb 13;24:200. doi: 10.1186/s12885-024-11934-2 (PMC10863204; doi:10.1186/s12885-024-11934-2)
Supplement: Supplementary file 1 — Additional file 1: Supplement figure 1. ROC curve of predictive score of overall population. Supplement figure 2. ROC curve of predictive score of EGFRm cohort. [file 12885_2024_11934_MOESM1_ESM.docx]

**Supplement Legend:**

Supplement figure 1: ROC curve of predictive score of overall population

Supplement figure 2: ROC curve of predictive score of *EGFRm* cohort

**Supplement figure 1: ROC curve of predictive score of overall population**

**Supplement figure 2: ROC curve of predictive score of *EGFRm* cohort**
